# Prevalence and Correlates of Depressive Symptoms among Patients with Cancer: A Cross-Sectional Study

**DOI:** 10.3390/curroncol31100431

**Published:** 2024-09-26

**Authors:** Wei-Zhen Yu, Hsin-Fang Wang, Nurul Huda, Yun Yen, Yen-Lin Liu, Chia-Sui Li, Yen-Chung Ho, Hsiu-Ju Chang

**Affiliations:** 1 School of Nursing, College of Nursing, Taipei Medical University, Taipei 110301, Taiwan; d432104005@tmu.edu.tw; 2 Nursing Department, Min-Sheng General Hospital, Taoyuan 330056, Taiwan; 3 Cancer Medical Center, Lo-Hsu Medical Foundation, Lotung Poh-Ai Hospital, Yilan 265501, Taiwan; 116096wang@gmail.com; 4 Nursing Faculty, Universitas Riau, Pekanbaru 28131, Indonesia; nurulmamaifda@gmail.com; 5 Program for Cancer Biology and Drug Discovery, College of Medical Science and Technology, Taipei Medical University, Taipei 110301, Taiwan; yyen@tmu.edu.tw; 6 Taipei Cancer Center, Taipei Medical University, Taipei 110301, Taiwan; ylliu@tmu.edu.tw; 7 Pediatric Oncology, Department of Pediatrics, Taipei Medical University Hospital, Taipei 110301, Taiwan; 8 Department of Pediatrics, School of Medicine, College of Medicine, Taipei Medical University, Taipei 110301, Taiwan; 9 Department of Community Medicine, En Chu Kong Hospital, New Taipei City 237414, Taiwan; m432106018@tmu.edu.tw; 10 Department of Nursing & Graduate Institute of Nursing, College of Nursing, Asia University, Taichung 413305, Taiwan; samuelho@asia.edu.tw; 11 College of Nursing, National Yang Ming Chiao Tung University, Taipei 112304, Taiwan; 12 Efficient Smart Care Research Center, College of Nursing, National Yang Ming Chiao Tung University, Taipei 112304, Taiwan

**Keywords:** nursing, cancer, demoralization, depression, distress, perceived benefits, anxiety, patient experience

## Abstract

The purpose of this study was to identify the correlates of depressive symptoms and the prevalence of depression, distress, and demoralization among patients with cancer in Taiwan in relation to their sociodemographics. A cross-sectional study design with convenience sampling was used to recruit 191 consecutive patients with cancer from the Cancer Center of a teaching hospital in northern Taiwan. Multiple linear regression was applied to analyze the determinants of depressive symptoms. The prevalence rates of depression (including suspected cases), distress, and demoralization were 17.8%, 36.1%, and 32.5%, respectively. The regression model explained 42.2% of the total variance, with significant predictors including marital status, life dependence, comorbidity, demoralization, and distress. The results demonstrated that higher levels of distress and demoralization were associated with more depressive symptoms. Demoralization and distress played vital roles in moderating depressive symptoms among patients with cancer. Nursing interventions should integrate appropriate mental health services, such as alleviating distress and demoralization, to prevent the occurrence of depression in patients with cancer.

## 1. Introduction

The International Agency for Research on Cancer estimates that in 2022 there were 20 million new cancer patients and 9.7 million cancer-related deaths worldwide [1]. A high proportion (25–35%) of patients with cancer also experience depression [2,3,4]. Cancer patients with comorbid psychiatric disorders are at higher risk of death compared to cancer patients without comorbidity, and they are less likely to receive stage-appropriate treatment [5]. Therefore, early identification of patients with the greatest risk of depression is essential to the early detection of mental health problems.

In Taiwan, depression among patients with cancer has been noted to particularly increase after diagnosis of the cancer, which highlights the importance of mental health care in cancer management [6]. Preventive strategies and integrating identification of the risks of mental disorders among patients with cancer into healthcare systems are key targets for improving mental health wellbeing [7]. Therefore, it is necessary to detect the risk of depression as early as possible and recognize the potential factors that affect depression, so as to subsequently prevent and ameliorate depression in cancer patients.

## 2. Background

Demoralization, distress, patient experience, perceived benefits, and anxiety are among the risk factors for psychological problems in patients with cancer, and they make such patients vulnerable to depression [6,7,8,9,10,11,12,13,14].

Demoralization is an existential distress syndrome that involves an incapacity to cope, feelings of hopelessness and helplessness, and a loss of meaning and purpose; it can also compromise one’s self-esteem [15]. Demoralization differs from depression in that patients who feel demoralized retain the ability to feel immediate happiness and more subjective incompetence, whereas those with depression do not. Specifically, the sense of incompetence associated with demoralization is caused by uncertainty about the appropriate direction of action, while depression lacks motivation even if the direction of action is known [16,17,18]. The prevalence rates of demoralization among patients with cancer reportedly range from 13.5% to more than 60% [16,19,20]. According to studies on patients with cancer in Germany (*n* = 516) by Mehnert et al. [21] and in Taiwan (*n* = 234) by Lee et al. [22] using the Depression Scale of PHQ-9 and the Demoralization Scale, both correlation matrices have positive associations (*r* = 0.61, *p* < 0.001; r = 0.617, *p* < 0.001, respectively). In addition to the above two articles, a literature review by Robinson et al. pointed out that nine other studies also showed a strong positive correlation between demoralization and depression. A recent systematic review of 36 studies by Wang et al. [19] found that the factors affecting depression associated with cancer are many and complex, including a strong correlation with suicidal ideation and an apparent intersection between demoralization and depression, suicidal ideation, and anxiety.

According to the definition of the NCCN [23], distress is “a multifactorial unpleasant experience of a psychological (i.e., cognitive, behavioral, emotional), social, spiritual, and/or physical nature… Distress extends along a continuum, ranging from common normal feelings of vulnerability, sadness, and fears to problems that can become disabling, such as depression, anxiety, panic, social isolation, and existential and spiritual crisis”. Distress occurs in patients with cancer mainly due to the lack of relevant medical information and inadequate professional treatment, leading to a high degree of uncertainty about the future, which in turn leads to anxiety and depression [11,13]. Distress encompasses anger, anxiety, and depressive symptoms [24]. The rate of distress in recent large-scale studies with more than 1000 people was 33.2% to 46.5% [25,26,27]. Depression has also been significantly related to distress [28,29]. McFarland et al. [29] studied 125 patients with breast cancer and found that the rate of depression (87%) among the physical problems (from the Physical Problem List) of distress was higher than the rates of anxiety (53%) or distress (27%).

The terms “patient experience” and “patient satisfaction” are often used interchangeably by researchers. Patient satisfaction is measured based on many factors falling under “patient experience” before, during, and after care, as well as characteristics of the care environment [30]. According to The Beryl Institute’s [31] definition, the patient experience is “the sum of all interactions, shaped by an organization’s culture, that influence patient perceptions across the continuum of care.” It is a multidimensional, multifaceted, and closely related concept and covers four key themes: personal interactions, organizational culture, patient and family perceptions, and continuum of care [32]. Patient experience therefore includes several aspects of healthcare on which patients place high value when seeking and receiving care, such as timely appointments, easy access to information, and good communication with healthcare providers [33]. According to a study by Bui et al. [10] of patients newly diagnosed with breast cancer (*n* = 210), each unit increase in depressive symptoms at baseline reduced the predicted odds of being “very satisfied” with medical care at follow-up by 6% (OR = 0.94, 95% CI = 0.89, 0.99). Satisfaction with medical care is negatively correlated with depression [34,35].

“Perceived benefits” are positive psychological changes patients observe in themselves following adversity, such as posttraumatic growth, stress-related growth, and thriving [36,37]. Because negative life experiences (adversity) in patients with cancer, including lower perceived social support and more stressful life events, are associated with depressive symptoms, perceived benefits following adversity may help prevent the onset of depression [38].

Regarding the evolution of anxiety comorbidity, research shows that anxiety disorders often precede the onset of major depression [39]. Interestingly, a study by Maass et al. [40] that included the results of 17 articles showed that depressive symptoms increased in the long term after breast cancer, but anxiety symptoms did not. A recent cross-sectional study surveyed 1011 cancer patients (399 inpatients and 612 outpatients) and found a significant correlation between anxiety and depression (r = 0.812) [41]. In addition, a systematic literature review of 40 studies indicated that four of these showed a positive correlation between anxiety and depression [7].

**Figure 1:**
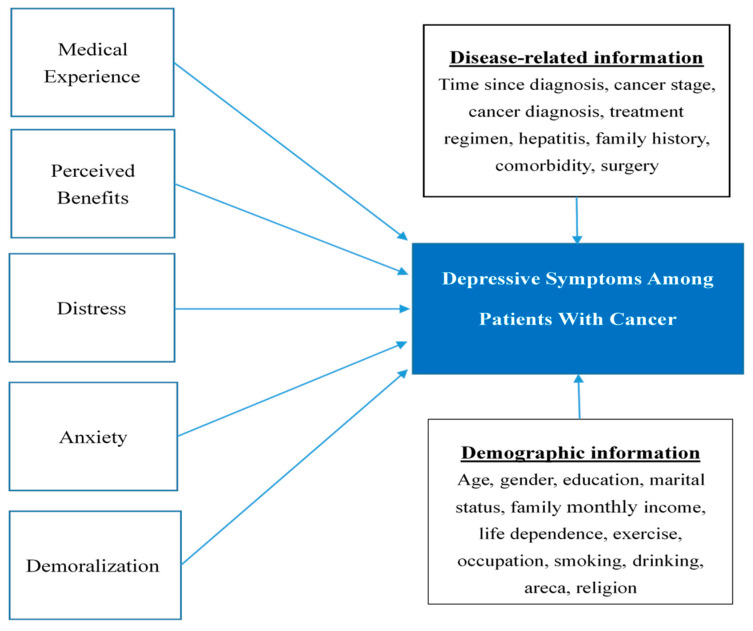
Conceptual framework.

Among these factors, to our knowledge, only demoralization has been extensively studied in Taiwanese populations with cancer [12,22,42,43,44]. However, other psychosocial elements that may lead to depressive somatic symptoms are just as important [45,46]. Hence, we hypothesize that demoralization, distress, perceived benefit, patient experience, and anxiety play important roles in cancer-related depression by influencing the subjective perception of situations and the consequent reactions to them. The conceptual structure of this study is shown in Figure 1. The main aim of this study was to investigate the correlates of depression. The prevalence of depression, distress, and demoralization, and the association of the latter two constructs with depression in patients with cancer, in relation to the patients’ sociodemographic information, were also examined.

## 3. Materials and Methods

### 3.1. Design

The study employed a cross-sectional design.

### 3.2. Instruments with Validity and Reliability

#### 3.2.1. Sociodemographic Information

Baseline demographic data, including age, gender, marital status, education level, occupation, time since diagnosis, household income, diagnosis, pathological stage, treatment plan, substance use (including tobacco, alcohol, and betel nut), history of hepatitis, family history, comorbidity, level of dependence for activities of daily living, exercise habits, and religion, were obtained. Patient medical records outlining cancer diagnosis and treatment were also reviewed.

#### 3.2.2. Distress

The Distress Thermometer (DT) is a validated screening tool for measuring psychological distress; it was endorsed by the NCCN [23]. The DT is a single-item self-reported questionnaire that is used to identify distress from any source. Participants were instructed to circle their answers on a scale of 0 (*no distress*) to 10 (*extremely distressed*) to indicate their level of distress in the preceding week. A score of ≥4 was considered the cutoff for a high distress level. The Chinese version of the DT, which included a Problem List, was employed. This version has demonstrated high sensitivity and specificity [47,48].

#### 3.2.3. Demoralization

The Demoralization Scale, Mandarin Version (DS_MV) was developed by Kissane et al. [49] and was translated into Chinese by Hung et al. [44]. This scale evaluates five aspects of demoralization: loss of meaning, dysphoria, disheartenment, helplessness, and a sense of failure. The scale contains 24 items scored using a five-point scoring method, with answers ranging from “*strong disagreement*” (0 points) to “*strong agreement*” (4 points). The cutoff score was set at 30 points, with a score of ≥30 points indicating demoralization. Cronbach’s alpha for the total scale was 0.928, and Cronbach’s alpha values for the individual aspects of the scale ranged from 0.63 to 0.88. The convergent and divergent validities were evidenced by a positive correlation with the Beck Hopelessness Scale (r = 0.703, *p* < 0.001) and a negative correlation with the McGill Quality of Life Questionnaire (r = −0.680, *p* < 0.001), respectively [44].

#### 3.2.4. Anxiety and Depression

Anxiety and depression were assessed using the Chinese version of the Hospital Anxiety and Depression Scale (HADS), which has been shown to have favorable reliability, specificity, and sensitivity [48,50]. The HADS is a self-administered questionnaire with two subscales of seven items each to evaluate levels of anxiety (HADS_A) and depression (HADS_D). Each item is scored from 0 to 3 points on a four-point Likert scale, with a higher score indicating a higher degree of anxiety or depression. A total score of less than 7, 8–10, and 11 or more respectively indicates that the patient has no anxiety or depression, has a suspected case of anxiety or depression, or has anxiety or depression [51]. In this study, a score ≥ 8 on the HADS_D questionnaire was considered to indicate depression.

#### 3.2.5. Perceived Benefits

The Perceived Benefit Scale was developed by McMillen and Fisher [52] and translated into Chinese by Wang [53]. The 38-item Chinese version contains five subscales: increased community closeness, enhanced family closeness, enhanced self-efficacy, increased spirituality, and increased compassion. The subscales are scored using a five-point Likert scale, ranging from 0 (*not at all like my experience*) to 4 (*very much like my experience*). Cronbach’s α for the subscales of the original scale ranged from 0.73 to 0.93, and for the Chinese version ranged from 0.76 to 0.86. The reliability of the original scale after two weeks ranged from 0.66 to 0.97. The Chinese version had expert and construct validity [53].

#### 3.2.6. Patient Experience

The Patient Assessment of Chronic Illness Care (PACIC) is a 20-item self-reporting instrument for assessing patients’ reception of clinical services and actions consistent with the Chronic Care Model, which was developed to ensure that patients receive care that is patient-centered, proactive, and well-planned and includes collaborative goal setting, problem solving, and follow-up. The PACIC has five subscales: patient activation, decision support, goal setting, problem solving, and follow-up or coordination. Cronbach’s α for the total scale was 0.93, and those for the subscales ranged from 0.77 to 0.90. The scale was demonstrated to have favorable construct validity and content validity [54].

### 3.3. Sampling and Recruitment

A convenience sampling method was used to recruit outpatients who were undergoing active cancer treatment, including surgery, radiotherapy, chemotherapy, hormonal therapy, targeted therapy, and immunotherapy, in a cancer center in northern Taiwan. Patients who met the following criteria were included: age ≥ 20 years, absence of a psychiatric diagnosis, ability to speak Chinese, and willingness to participate in the study and provide written informed consent.

### 3.4. Sample Size and Power

In establishing the sample size, we considered α = 0.05, β = 0.2, the medium effect size = 0.25, and a 20% dropout rate. Of the 472 qualifying patients, a total of 191 were included in the sample, which fulfilled the minimum required sample size of 154 as estimated using G Power software version 3.1.

### 3.5. Data Sources/Collection

Patients were recruited through convenience sampling conducted from January 2019 to January 2020. Before recruiting patients during their outpatient visits, the principal investigator first discussed the study with the physicians of the cancer center and obtained their consent. Researchers then screened for patients who met the criteria for inclusion by searching the hospital outpatient information system for patients with qualifying medical-related information.

Initial contact with patients was made via telephone, during which the researchers provided an overview of the study’s purpose, procedures, and benefits. Patients who expressed interest in participating were then scheduled for a face-to-face meeting with the researchers during their outpatient visit. During this meeting, the researchers provided a detailed explanation of the study, including relevant benefits, potential risks, data processing, and utilization. Participants were given ample opportunity to ask questions and clarify any concerns. Trained researchers were available to assist and ensure participants fully understood the study before they signed the informed consent form and completed the study questionnaires. However, the data collection process was thoroughly documented as part of the overall research plan, with specific details available in the publication by Yu et al. [55].

### 3.6. Data Analysis

Data were analyzed using SPSS Statistics for Windows, version 24 (IBM, Armonk, NY, USA). Missing values were handled by pairwise deletion. Descriptive statistics and frequency distributions were calculated for the lists of sociodemographic and clinical characteristics. The *t*-test and analysis of variance were performed to determine differences in sociodemographic and disease-related variables with respect to depression. The prevalence of distress, anxiety, depression, and demoralization was determined using descriptive statistics and frequency analysis. Pearson correlation analysis was conducted to identify potential associations among age, time since diagnosis, distress, anxiety, depression, demoralization, perceived benefits, and patient experience. Finally, multiple linear regression was used to identify the determinants of depressive symptoms. The dependent variable (depressive symptoms) was a continuous variable. The independent variables were selected based on the statistical method proposed by Bursac et al. [56]. Factors that showed significant results in the chi-squared tests, t-tests, and Pearson correlation analyses of the aforementioned sociodemographic and clinical characteristics were included in the multiple linear regression model. This approach was used to analyze the factors influencing depressive symptoms, thereby avoiding potential issues such as model instability, inflated results, or unreasonable outcomes that could arise from including categorical variables with small sample sizes or too many variables with minimal influence. Categorical variables were converted into dummy variables for analysis. The enter method was used for the multiple linear regression analysis. A *p*-value < 0.05 was considered to indicate statistical significance.

### 3.7. Ethical Considerations

The study was approved by the Joint Institutional Review Board of a medical university in northern Taiwan (IRB number N201809031). Written informed consent was obtained from all participants. The informed consent process involved providing participants with detailed information about the study’s purpose, procedures, potential risks, and benefits, as well as their right to withdraw from the study at any time without any consequences. Consent forms were signed by the participants and were securely stored in a locked file cabinet accessible only to authorized research personnel. All participants were provided with a copy of the consent form for their records.

## 4. Results

### 4.1. Sociodemographic and Disease-Related Information

#### 4.1.1. Demographic Information

Overall, 191 out of the 472 qualifying patients completed the questionnaires, indicating an overall response rate of 40.5%. The main reason for refusal to participate was not having time to complete the study questionnaires. The mean age of patients was 55.3 ± 12.04 years. As indicated in Table 1, the participants were primarily women (73.3%) and married (62.8%), and seven out of ten patients reported religious affiliations. Among the patients with cancer, higher levels of depressive symptoms were experienced by males and those with a household income per month of less than TWD$ 29,999 and within the range of TWD$ 70,000 to 89,999; smokers; those who were separated or divorced; and those with a high level of dependence in life. However, the Scheffé test did not reveal significant differences among patients with different household monthly incomes.

#### 4.1.2. Disease-Related Information

Stage 0 refers to cancer in situ that has not spread [57,58]. Most of the respondents were in stage I (25.7%) of their cancer, followed by stage II (25.1%), stage IV (25.1%), stage III (15.7%), and stage 0 (8.4%). Regarding tumor type, almost half of the patients (53.4%) had breast cancer, followed by other cancers (23.6%) (such as prostate, oral, gynecological, lymphoma, and liver), colorectal (11.5%), lung (5.8%), and gastrointestinal/pancreatic cancer (5.8%). Regarding treatment, 40% of the patients were undergoing more than two treatment plans, whereas just 2.1% were undergoing forms of treatment other than chemotherapy, radiotherapy, hormone therapy, and target therapies. Chemotherapy was the preferred treatment (31.4%). Furthermore, almost half of the patients (49.7%) had a family history of cancer, and more than a third (38.7%) had comorbidity; 13.6% of patients had hepatitis. The disease-related information is presented in Table 2. Among the patients with cancer, higher levels of depressive symptoms were experienced by those with more comorbidity. Patients who were given diagnoses of gastric or pancreatic cancer experienced the highest level of depressive symptoms. However, the Scheffé test did not reveal significant differences among patients given different cancer diagnoses.

### 4.2. Prevalence of Depressions, Anxiety, Demoralization, and Distress

The mean HADS_D, HADS_A, DS_MV, and DT scores were 4.74 (SD = 3.00), 5.1 (SD = 3.08), 24.71 (SD = 12.51), and 2.96 (SD = 2.34), respectively. The prevalence rates of depression, anxiety (including suspected), and demoralization were 17.8%, 19.4%, and 32.5%, respectively. In addition, 36.1% of participants (69/191) experienced a high level of distress, and 4.97% (9/191) were highly vulnerable patients with scores that met the cutoff points for the HADS_D (including suspected), HADS_A (including suspected), DS_MV, and DT. The mean HADS_D score was 9.56 (SD = 2.46). The results are presented in Table 3.

### 4.3. Correlations between Age, Time since Diagnosis, and Study Variables

The correlations between age, time since diagnosis, and the study variables are presented in Table 4. Depression was significantly and positively associated with anxiety (r = 0.34, *p* < 0.001), psychological distress (r = 0.38, *p* < 0.001), and demoralization (r = 0.57, *p* < 0.001). By contrast, depression was significantly and negatively associated with perceived benefit (r = −0.26, *p* < 0.001) and patient experience (r = −0.19, *p* = 0.007). No significant correlation was identified between age and time since diagnosis (r = 0.08/0.04, *p* > 0.05).

### 4.4. Analysis of Determinants of Depressive Symptoms

Based on the results of the previous difference analysis in Table 1 and Table 2, the significant variables identified were gender, marital status, smoking, life dependence, comorbidity, and the scores of major indicators (Anxiety Scale, Depression Scale, Distress Thermometer, Perceived Benefit Scale, and Patient Medical Experience Scale). These variables were included in the multiple linear regression model analysis with the dependent variable being the Depression Scale score. The model indicated that current smoking habits, life dependence, comorbidity, distress, and demoralization are important determinants of depressive symptoms. The results are summarized in Table 5.

Regarding psychological predictors, after adjusting for basic demographic and disease-related variables, for each standard deviation increase in depression (DS_MV score), the depression score increased by 0.303 standard deviations (95% CI for B = 0.035–0.110, *p* < 0.001). For each standard deviation increase in distress (DT score), the depression score increased by 0.142 standard deviations (95% CI for B = 0.011–0.353, *p* = 0.037). Therefore, among all predictors, demoralization (DS_MV score) had the greatest impact. After adjusting for basic variables including smoking habits, life dependence, comorbidity, and distress, each one-unit (point) increase in demoralization (DS_MV score) resulted in a 0.073-point increase in the depression score. After adjusting for basic variables such as smoking habits, life dependence, comorbidity, and demoralization, each one-unit (point) increase in distress (DT score) resulted in a 0.182-point increase in the depression score. Collectively, these independent variables (Marital status_single, Marital status_married, life dependence, comorbidity, distress, and demoralization) explained 42.2% of the variance in depression.

## 5. Discussion

### 5.1. Main Purpose and Findings

The main purpose of this study was to explore the correlates of depressive symptoms among patients with cancer in Taiwan. It also investigated the prevalence of depression, demoralization, and distress among patients with cancer in Taiwan as well as the sociodemographic characteristics and disease-related factors that affect the prevalence of depression. The results revealed that the prevalence of distress (36.1%) among patients with cancer was higher than that of depression (17.8%) and demoralization (32.5%). Marital status, life dependence, and comorbidity were significant predictors of depression, with higher levels of distress and demoralization correlating with increased depressive symptoms.

### 5.2. Comparison with Previous Studies 

The findings of this study indicate that the rate of depression (including suspected depression) among patients with cancer in Taiwan is approximately 17.8%. However, a study in Australia reported the depression rate among patients with cancer to be 45% [59]. This rate is much higher than that calculated in our study. The discrepancy may be due to differences in disease characteristics, types of cancer treatment, number of side effects of cancer treatment, and assessment methods [60]. In addition, cultural differences may have influenced the reported incidence of depressive symptoms among patients with cancer. Compared with the personal orientation of Europe and the United States, Chinese people are more family-oriented and less revealing of their feelings and taboos about death [61,62]. Asian patients often focus more on physical rather than psychological symptoms, and the depressive symptoms may have been underestimated in our study [63].

The prevalence of demoralization in our study was 32.5%, which was higher than the global prevalence of 13–18% found in the systematic review by Robinson et al. [17]. Their study used PRISMA guidelines to search nine electronic bibliographic databases and found 33 articles from 2000 to 2013, with a total of 4545 participants from Australia, the United States, Italy, Taiwan, Hungary, Germany, Japan, Ireland, and Portugal. The prevalence of demoralization may be driven by the influence of sociodemographic and psychological factors. Research indicates that older age is associated with higher scores for demoralization [16]. In addition, patients with cancer who reported being unemployed had higher scores for demoralization [17]. The majority of our participants were women, older adults, and were unemployed; these demographic factors likely led to the prevalence of demoralization being higher in our study than in others. Furthermore, demoralization is related to psychological factors; patients with cancer who experience depression are prone to demoralization [17]. Given that our participants had higher levels of depression, the higher prevalence of demoralization in our study is unsurprising.

Regarding anxiety, this study found a prevalence rate of 19.4% (including suspected cases), which is consistent with the 19.1% rate reported by Naser et al. [41] at the Amman Cancer Center, despite their study excluding suspected anxiety cases. The higher anxiety prevalence in our study may be attributed to the specific cancer types in our population, as nearly two-thirds were diagnosed with lung, breast, prostate, or head and neck cancers—types known to be associated with higher anxiety levels [46]. Similarly, Goerling et al. [64] reported a 13.8% anxiety prevalence in a German multicenter study using the GAD-7, with particularly high anxiety risks in bladder and testicular cancer patients (OR, 5.3; 95% CI = 3.0–9.4). The differences in cancer types and our smaller sample size limit direct comparisons, but these findings align with our observation of elevated anxiety levels in specific cancer populations. Additionally, a meta-analysis incorporating 36 studies and 16,298 breast cancer patients reported an anxiety prevalence rate of 41.9% (95% CI = 30.7–53.2), underscoring the high prevalence of anxiety in this population. The study highlighted the significant impact of psychological factors on anxiety levels among breast cancer patients, emphasizing the importance of addressing these factors in clinical care [65].

Distress is a common mental health problem that occurs among patients with cancer. In this study, almost a third of the participants experienced distress. However, a study from Australia found that almost 91% of patients with cancer reported clinically significant stress, indicating that the prevalence in that study was three times higher than that in our study. The discrepancy may be related to cultural values. People from Western countries were found to seek support when they perceived information or treatment to be insufficient, rather than continue to complete the routine procedure, which may explain why they report distress more frequently than Asian people do [13,66].

In this study, the prevalence of chronic comorbidities among cancer patients was 38.7%. Generally, cancer patients tend to have more physical comorbidities compared to those without a history of cancer. According to Petrova et al. [67], who analyzed 484 patients in Spain diagnosed with cancer within a year, the prevalence of chronic comorbidities was 89.7%. Furthermore, each additional comorbidity increased the likelihood of psychological distress (GHQ-12) by 9% (OR = 1.09, 95% CI = 1.01–1.16). Another study in the United States involving 2073 cancer patients found a significant association between the number of comorbidities at the time of cancer diagnosis and the risk of recent depression (PHQ-9), with those having multiple comorbidities being 3.48 times more likely to exhibit significant depressive symptoms within five years compared to those without comorbidities (95% CI = 1.26–9.55). The most impactful comorbidities were stroke, kidney disease, hypertension, obesity, asthma, and arthritis [68]. Additionally, a study in China with 1546 participants found that cancer patients with one or two chronic comorbidities were 1.35 times more likely to suffer from depression (95% CI = 1.04–1.75, *p* = 0.022), and those with three or more comorbidities were 1.74 times more likely (95% CI = 1.30–2.33, *p* < 0.001) [69]. The primary difference between these studies and the current research is the stage of patients; while previous studies focused on post-treatment patients, this research involves patients in various stages of treatment. Nevertheless, comorbidities consistently emerge as predictors of depressive symptoms.

Numerous studies have shown that as cancer patients’ self-care abilities improve, their dependence on others is reduced, leading to a reduction in depressive symptoms [70,71,72]. Self-care ability and dependence on others are inversely related [73,74]. When self-care ability declines, dependence on others increases correspondingly [74]. Due to mobility impairments caused by the disease, cancer patients often rely on others for daily care, which diminishes their independence and sense of control, contributing to depression. Additionally, the guilt associated with requiring care and the financial burdens they face can further exacerbate emotional distress, making depressive symptoms more likely [72,74,75]. This study also found that life dependence is a significant factor influencing depression symptoms. People who are dependent on others and unable to care for themselves exhibit more severe depression problems than those who maintain independence.

The results of the present study indicate that women tended to have lower levels of depression than men. However, another study reported lower depression levels in men [76]. A recent study in Taiwan found that men with prostate cancer had higher anxiety and depression problems, which may be due to the cultural phenomenon that men value “face” and dignity more than women, suppress emotions, and limit social support [77]. Furthermore, this could be explained by the greater age of most men in our study. Older patients experience depression more often because they experience multiple losses in their lives over a short period. In addition to experiencing declining health due to cancer and age, many older patients experience the loss of a spouse, functional impairment, and a poor social network, which leads them to feel more depressed [78,79,80,81]. They also experience lower levels of concentration and difficulty in remembering, which prevent them from recognizing that they have experienced symptoms of depression and lead them to delay seeking help.

The results of this study indicate that smoking was associated with a higher risk of depression. According to the self-medication model, people smoke because they want to alleviate psychological symptoms [82]. When these people experience symptoms of depression, they attempt to minimize them by smoking. However, the relationship between smoking and depression is bidirectional, which indicates that prolonged smoking can also perpetuate depression [82]. Choi and Park [83] conducted a study in Korea involving 1163 cancer survivors and found that smokers had a 1.7 times higher risk of depression compared to non-smokers (OR = 1.73, 95% CI = 1.04–2.87).

In this study, having an “other” marital status, such as being separated or divorced, was revealed to be a risk factor for depression. Similar to a previous study [84], the prevalence of depression among widows who were diagnosed with cancer was found to be higher than that of married patients. Patients who are separated or divorced may not have someone with whom they can share emotions, thoughts, or decisions or who can provide them with support for treatments and healthcare visits. Therefore, when patients lose support, they are at a higher risk of developing depression. The results indicate that patients with colorectal cancer and other types of cancer were more likely to have depression compared to those with breast cancer. This is in line with the findings reported in a previous study [2]. Patients with breast cancer are usually mildly depressed and may have improved family support, self-care, patient satisfaction, and medical care related to the improvement of quality of life [66,85].

This study demonstrated that distress and demoralization significantly affect depression. This is in line with the findings of Robinson et al. [17], who demonstrated that demoralization can be a risk factor for or a prodromal symptom of depression. This could explain why most patients with cancer who felt demoralized had similar symptoms to those of depression, such as feelings of sadness and a loss of hope. The prolonged loss of enjoyment resulting from experiencing cancer may be associated with a loss of hope and a feeling that life is no longer worth living, which may precede depression [16].

The results of the present study suggest that distress can increase the risk of depression. The risk of depression is commonly believed to significantly increase when distress levels are higher, which can affect a patient’s quality of life [86]. However, Ng et al. [87] reported no association between any level of distress and depression. Their results may be explained by the complexity of depression among patients with cancer, with the symptoms often varying in clinical presentation, leading to some features potentially being underestimated or overestimated. This variation often leads to clinicians misidentifying symptoms of depression, which may explain why some studies have identified an association between distress and depression while others have not. Furthermore, this relationship may be mediated by patients’ strategies for coping with their illness trajectories [86], although in the current study, we did not explore coping strategies. Further investigation may be warranted to clarify this potential relationship.

This study revealed that perceived benefits, including aspects related to social support such as increased community and family closeness, could serve as a protective factor against depression. People often report having benefited from negative life events, like a cancer diagnosis [52], which can accelerate patients’ recovery and enhance psychosocial functioning. However, the results did not show a significant impact of perceived benefits on depressive symptoms. Despite this, social support remains a crucial aspect of mental health for cancer patients. While perceived benefits as a whole might not have significantly influenced depression in our sample, specific elements of social support could still play a vital role.

The regression model employed in our study explained 42.2% of the total variance. According to previous reports, personal health behaviors (physical activity or smoking habits), social status (low degree of social support such as being separated or divorced, and life dependence), and psychological status (demoralization and distress) can reflect psychological morbidity more accurately than can disease characteristics [88,89]. In the current study, sociodemographic and psychological factors played key roles in influencing the development of depression among patients with cancer.

Although previous literature and clinical observations suggest that different cancer types, such as breast cancer, might influence depressive symptoms due to their unique biopsychological contexts, our analysis did not find cancer type to be a significant predictor. A Scheffé post-hoc test showed no significant differences in depressive symptoms among the cancer type. Even after including cancer type in the multiple linear regression model (as shown in Supplementary Table S1), it remained non-significant. This result suggests that, within our study population, depressive symptoms are more strongly influenced by factors such as psychological distress and demoralization rather than the specific type of cancer. This finding underscores the importance of focusing on psychosocial factors when addressing depression in cancer patients, rather than assuming that cancer type alone will dictate the psychological outcomes.

### 5.3. Limitations of the Work

Several limitations should be considered when interpreting the results of this study. First, the use of convenience sampling may introduce selection bias, as certain characteristics of the participants may be overrepresented or underrepresented. Second, while we initially considered cancer type as a potential predictor of depressive symptoms, it was excluded from the final regression model due to its lack of significant impact. This exclusion suggests that cancer type may not significantly influence depressive symptoms within our sample. However, this finding may not be generalizable to larger or more diverse populations, where cancer type could play a more significant role. The absence of a significant effect may also reflect the specific characteristics of our sample rather than a universal finding, indicating that future studies with larger sample sizes and diverse cancer types are needed to clarify the role of cancer type in the psychological outcomes of cancer patients. Additionally, the study did not account for biological mechanisms that might induce depression, such as those activated by medications used to alleviate cancer treatment side effects. This omission limits our understanding of the potential interplay between biological and psychological factors in the development of depressive symptoms. Moreover, the reliance on self-reported questionnaires could have led to an overestimation of the prevalence of depression, as patients might have exaggerated or understated their symptoms, affecting the accuracy of the data collected. Furthermore, because participants in this study were recruited from a single referral hospital in northern Taiwan, the findings may not be generalizable to other populations. The cultural context of Taiwan, including specific attitudes toward mental health and the experience of illness, might also have influenced how patients reported and perceived depressive symptoms. In particular, there is a cultural tendency among some patients to self-attribute their cancer diagnosis to bad living habits and irresponsibility for their own health [90]. This self-blame may exacerbate feelings of guilt and shame, potentially influencing how they experience and report depressive symptoms. Such cultural factors could contribute to variations in how depression is recognized and addressed among cancer patients in Taiwan, complicating the interpretation of the study’s findings.

### 5.4. Recommendations for Further Research

To address the study’s limitations and build on its findings, future research should employ random sampling to improve representativeness and generalizability. Incorporating biological markers and treatment variables will enhance our understanding of how biological mechanisms contribute to depression in cancer patients. Since our findings indicated that cancer type did not significantly impact depressive symptoms within our sample, future studies should explore this further in larger, more diverse populations to determine if this result holds true in different contexts. Detecting patients’ distress and demoralization can help identify the likelihood of these patients suffering from depression and slow the progression of depression. Regular assessments of these underlying factors could provide valuable insights into the mental health trajectory of cancer patients. Additionally, integrating clinical assessments or longitudinal follow-ups with self-reported data would allow for more accurate estimates of depression prevalence, reducing potential biases. A multidisciplinary approach to nursing interventions could be designed to assess the reduction in distress-related maladaptive outcomes. Furthermore, real-time services via telephone and dedicated internet lines can also be provided to establish good communication channels, allowing cancer patients to easily obtain medical care-related information and assist them with medical treatment and adjustment at home. Finally, future research should also more directly investigate the impact of social support, potentially isolating it from broader constructs like perceived benefits, to better understand its moderating effects on depression among cancer patients [9].

## 6. Conclusions

The prevalence of depression among the Taiwanese population with cancer is similar to global prevalence. Depression was influenced by distress, demoralization, and perceived benefits. Patients with higher levels of distress and demoralization were more vulnerable to developing depression, whereas higher perceived benefits were a protective factor that led patients with cancer to be less prone to developing depression. These findings expand upon our understanding of the role of demoralization and psychological distress in predicting depressive symptoms in patients with cancer. Nursing interventions should integrate appropriate mental health services to prevent negative outcomes of depression in patients with cancer.

## Figures and Tables

**Table 1:** Descriptive statistics for demographics and differences in HADS_D (*N* = 191).

	Number (*n*)/Mean (SD)	Percent (%)	HADS_D (Mean)	HADS_D (SD)	*p*-Value/ Statistics
Age	55.3 (12.04)	-	-	-	-
Gender					
Male	51	26.7	5.75	3.155	*p* = 0.005
Female	140	73.3	4.38	2.865	t = 2.837
Marital status					
Single	47	24.6	4.04	2.621	*p* = 0.001 F = 7.766 post hoc: single vs. other *p* = 0.010 married vs. other *p* = 0.036
Married	120	62.8	4.6	2.748	
Other (separated, divorced)	24	12.6	6.83	3.975	
Occupation					
No	101	52.9	4.95	3.339	*p* = 0.306
Yes	90	47.1	4.51	2.563	t = 1.026
Education					
≤Primary school (9 grade)	31	16.2	5.45	3.793	*p* = 0.541 F = 0.720
High school	54	28.3	4.63	3.036	
College graduate	90	47.1	4.56	2.805	
Postgraduate degree	16	8.4	4.81	2.136	
Family monthly income (TWD) (N = 181)					
≤29,999	33	17.3	5.52	3.607	*p* = 0.036 F = 2.639 post hoc: (Scheffé) non-significant
30,000–49,999	38	19.9	3.71	2.680	
50,000–69,999	43	22.5	4.86	2.900	
70,000–89,999	19	9.9	5.74	2.557	
≥90,000	48	25.1	4.25	2.733	
Smoking					
No	145	75.9	4.32	2.784	*p* = 0.001
Yes ^a^	46	24.1	6.07	3.289	t = 3.533
Drinking					
No	99	51.8	4.81	3.212	*p* = 0.757
Yes	92	48.2	4.67	2.766	t = 0.310
Areca (betel nut)					
No	173	90.6	4.62	2.872	*p* = 0.074
Yes	18	9.4	5.94	3.918	t = −1.796
Life dependence					
No	173	90.6	4.45	2.699	*p* = 0.005
Yes	18	9.4	7.61	4.146	t = −3.170
Exercise					
No	61	31.9	5.28	3.312	*p* = 0.101
Yes	129	67.5	4.47	2.812	t = 1.657
Religion					
No	54	28.3	4.96	3.040	*p* = 0.527
Yes	137	71.7	4.66	2.989	t = 0.634

Abbreviations: HADS_D, Hospital Anxiety and Depression Scale–Depression; ^a^ “Smoking:Yes” refers to occasional smoking (only in social situations, never alone), current smoking (smoking addiction), and abstinence.

**Table 2:** Differences in disease-related information (*N* = 191).

Characteristic	Number (%)	HADS_D (Mean ± SD)	Statistics, *p*-Value
Time since diagnosis			
≤1 year	129 (67.5)	4.53 ± 3.03	t = −1.390, *p* = 0.166
>1 year	62 (32.5)	5.23 ± 2.90	
Cancer stage			
0	16 (8.4)	3.63 ± 3.38	F = 1.762, *p*= 0.138
I	49 (25.7)	4.55 ± 2.72	
II	48 (25.1)	4.6 ± 2.76	
III	30 (15.7)	4.47 ± 3.18	
IV	48 (25.1)	5.63 ± 3.16	
Cancer Diagnosis			
Colorectal	22 (11.5)	5.59 ± 3.23	F = 3.161, *p*= 0.015 post hoc: no significant (Scheffé)
Lung	11 (5.8)	5.55 ± 2.58	
Breast	102 (53.4)	4.04 ± 2.63	
Gastric & pancreatic	11 (5.8)	5.64 ± 4.32	
Others (prostate, oral, gynecological, lymphoma, liver)	45 (23.6)	5.51 ± 3.12	
Treatment regimen			
Chemotherapy	60 (31.4)	5.17 ± 3.13	F = 2.149, *p* = 0.062
Radiotherapy	38 (19.9)	3.84 ± 2.69	
Hormone	26 (13.6)	3.77 ± 2.03	
Target	23 (12)	5.78 ± 3.64	
More than two types	40 (20.9)	5 ± 2.96	
Others (surgery, folk medicine)	4 (2.1)	4.75 ± 3.30	
Hepatitis			
No	165(86.4)	4.61 ± 2.95	t = −1.530, *p* = 0.128
Yes	26 (13.6)	5.58 ± 3.25	
Family history			
No	83 (43.5)	4.89 ± 3.02	F = 1.159, *p* = 0.316
Unknown	13 (6.8)	3.54 ± 2.82	
Yes	95 (49.7)	4.78 ± 2.99	
Comorbidity			
No	117 (61.3)	4.36 ± 2.73	t = −2.252, *p* = 0.025
Yes	74 (38.7)	5.35 ± 3.30	
Surgery (n = 190)			
No	34 (17.8)	5.44 ± 2.92	t = 1.47, *p* = 0.143
Yes	156 (82.1)	4.61 ± 3.01	

Abbreviations: HADS_D, Hospital Anxiety and Depression Scale–Depression.

**Table 3:** Scores and cutoff points for depression, anxiety, demoralization, and distress (*N* = 191).

	Number (%)	Mean ± SD
HADS_D		4.74 ± 3.00
Non-depression (<8)	157 (82.2)	3.73 ± 2.12
Suspected (8–10)	26 (13.6)	8.58 ± 0.86
Depression (≥11)	8 (4.2)	12.13 ± 1.36
HADS_A		5.1 ± 3.08
Non-anxiety (<8)	154 (80.6)	4.11 ± 2.21
Suspected (8–10)	29 (15.2)	8.76 ± 0.83
Anxiety (≥11)	8 (4.2)	12.88 ± 1.64
DT degree		2.96 ± 2.34
<4	122 (63.9)	1.45 ± 1.12
≥4	69 (36.1)	5.62 ± 1.34
DS_MV degree		24.71 ± 12.51
<30	129 (67.5)	18.11 ± 8.19
≥30	62 (32.5)	38.45 ± 7.91
HADS_D ≥ 8, HADS_A ≥ 8, DT ≥ 4, and DS_MV ≥ 30 ^a^	9 (4.97)	9.56 ± 2.46 (for HADS_D)

Abbreviations: HADS_A, Hospital Anxiety and Depression Scale–Anxiety; DT, Distress Thermometer; DS_MV, Demoralization Scale–Mandarin Version; ^a^ Scores of all four scales are greater than or equal to the cutoff point.

**Table 4:** Summary of Pearson correlation coefficients between age, time since diagnosis, HADS_D, HADS_A, PBS, DT, and PACIC.

	Age	Time Since Diagnosis	HADS_D	HADS_A	DT	DS_MV	PBS	PACIC
Age	1							
Time since diagnosis	0.06	1						
HADS_D	0.08	0.04	1					
HADS_A	−0.17 *	0.10	0.34 ***	1				
DT	−0.14	0.06	0.38 ***	0.51 ***	1			
DS_MV	0.02	0.03	0.57 ***	0.54 ***	0.46 ***	1		
PBS	0.23 **	0.03	−0.26 ***	−0.18 *	−0.15 *	−0.40 ***	1	
PACIC	0.05	−0.1	−0.19 **	−0.16 *	−0.15 *	−0.23 **	0.25 ***	1

Abbreviations: HADS_D, Hospital Anxiety and Depression Scale–Depression; HADS_A, Hospital Anxiety and Depression Scale–Anxiety; DT, Distress Thermometer; DS_MV, Demoralization Scale, Mandarin Version; PBS, Perceived Benefits Scale; PACIC, Patient Assessment of Chronic Illness Care; * *p <* 0.05, ** *p* < 0.01, *** *p* < 0.001.

**Table 5:** Potential variables associated with depression in patients with cancer.

Potential Variables		Univariate Regression Analysis					Multiple Regression Analysis			
	B	95% CI for B		β	*p*	B	95% CI for B		β	*p*
Age	0.021	−0.015	0.057	0.084	0.248	0.006	−0.026	0.039	0.025	0.709
Gender_male	1.367	0.417	2.317	0.202	0.005	0.784	−0.181	1.749	0.116	0.111
Smoking	1.741	0.769	2.713	0.249	0.001	0.689	−0.268	1.646	0.099	0.157
Life dependence	3.166	1.769	4.563	0.309	<0.001	1.915	0.745	3.085	0.187	0.001
Marital status_single (vs. other)	−2.791	−4.225	−1.357	−0.402	<0.001	−1.670	−2.866	−0.474	−0.240	0.006
Marital status_married (vs. other)	−2.233	−3.511	−0.955	−0.361	0.001	−1.546	−2.594	−0.498	−0.250	0.004
Comorbidity	0.992	0.123	1.862	0.162	0.025	0.865	0.131	1.598	0.141	0.021
DT	0.489	0.319	0.658	0.382	<0.001	0.182	0.011	0.353	0.142	0.037
DS_MV	0.136	0.107	0.164	0.565	<0.001	0.073	0.035	0.110	0.303	<0.001
PBS	−0.047	−0.072	−0.022	−0.264	<0.001	−0.011	−0.034	0.012	−0.061	0.344
Anxiety	0.330	0.199	0.462	0.339	<0.001	0.084	−0.056	0.223	0.086	0.237
PACIC	−0.609	−1.052	−0.166	−0.193	0.007	−0.155	−0.515	0.206	−0.049	0.398

Abbreviations: CI, Confidence Interval; vs., versus.

## Data Availability

Data from our study are unavailable due to privacy or ethical restrictions.

## Supplementary Materials

The following supporting information can be downloaded at: https://www.mdpi.com/article/10.3390/curroncol31100431/s1, Table S1: Multiple linear regression analysis of depression in cancer patients, including cancer type.
