# Supplementary material for: Prevalence and Correlates of Depressive Symptoms among Patients with Cancer: A Cross-Sectional Study
Source: Curr Oncol. 2024 Sep 26;31(10):5802–20. doi: 10.3390/curroncol31100431 (PMC11506764; doi:10.3390/curroncol31100431)
Supplement: Supplementary file 1 [file curroncol-31-00431-s001.zip › curroncol-3092927-supplementary.pdf]

Table S1. Multiple linear regression analysis of depression in cancer patients, including cancer type.

potential variables	Unstandardized Coefficients		Standardized Coefficients	95% CI for B		t	*p*
	**B**	**SE**	**β**	**Lower**	**Upper**		
Constant	3.449	1.450		0.588	6.310	2.380	0.018
Age	0.005	0.017	0.021	-0.028	0.038	0.317	0.752
Gender _ male	0.893	0.578	0.132	-0.248	2.035	1.545	0.124
Smoking	-1.628	0.614	-0.235	-2.841	-0.416	-2.650	0.009
Life dependence	-1.560	0.539	-0.252	-2.624	-0.495	-2.891	0.004
Marital status _ single (vs others [a])	0.612	0.498	0.088	-0.371	1.596	1.229	0.221
Marital status_ married (vs others [a])	0.874	0.379	0.142	0.126	1.622	2.306	0.022
Comorbidity	1.965	0.612	0.192	0.757	3.174	3.211	0.002
Colorectal cancer (vs breast cancer)	-0.415	0.631	-0.044	-1.660	0.831	-0.657	0.512
Lung cancer (vs breast cancer)	0.364	0.811	0.028	-1.237	1.965	0.448	0.655
Gastric & pancreatic cancers (vs breast cancer)	0.133	0.819	0.010	-1.484	1.749	0.162	0.872
Others [b] (vs breast cancer)	-0.102	0.521	-0.015	-1.131	0.927	-0.196	0.845
DT	0.183	0.089	0.143	0.008	0.358	2.065	0.040
DS_MV	0.075	0.020	0.312	0.036	0.113	3.836	0.000
PBS	-0.011	0.012	-0.064	-0.035	0.012	-0.977	0.330
Anxiety	0.082	0.072	0.085	-0.060	0.224	1.145	0.254
PACIC	-0.150	0.185	-0.048	-0.515	0.214	-0.814	0.417

potential variables	Univariate Regression Analysis					Multiple Regression Analysis				
	**B**	**95.0% CI for B**		**β**	***p***	**B**	**95.0% CI for B**		**β**	***p***
Age	0.021	-0.015	0.057	0.084	0.248	0.006	-0.026	0.039	0.025	0.709
Gender_male	1.367	0.417	2.317	0.202	0.005	0.784	-0.181	1.749	0.116	0.111
Smoking	1.741	0.769	2.713	0.249	0.001	0.689	-0.268	1.646	0.099	0.157
Life dependence	3.166	1.769	4.563	0.309	<0.001	1.915	0.745	3.085	0.187	0.001
Marital status_single	-2.791	-4.225	-1.357	-0.402	<0.001	-1.670	-2.866	-0.474	-0.240	0.006
Marital status_married	-2.233	-3.511	-0.955	-0.361	0.001	-1.546	-2.594	-0.498	-0.250	0.004
Comorbidity	0.992	0.123	1.862	0.162	0.025	0.865	0.131	1.598	0.141	0.021
DT	0.489	0.319	0.658	0.382	<0.001	0.182	0.011	0.353	0.142	0.037
DS_MV	0.136	0.107	0.164	0.565	<0.001	0.073	0.035	0.110	0.303	< 0.001
PBS	-0.047	-0.072	-0.022	-0.264	<0.001	-0.011	-0.034	0.012	-0.061	0.344
Anxiety	0.330	0.199	0.462	0.339	<0.001	0.084	-0.056	0.223	0.086	0.237
PACIC	-0.609	-1.052	-0.166	-0.193	0.007	-0.155	-0.515	0.206	-0.049	0.398

Note: a separated, divorced; b prostate and urological cancers, oral (including nasopharyngeal) cancer, gynecological cancer, lymphoma, liver cancer, esophageal cancer, gynecological cancers (including uterine and ovarian cancer), osteosarcoma; Abbreviations: SE, Standard. Error; CI, Confidence Interval.
